# A survey of ictal physical examination during VEEG monitoring in a tertiary epilepsy center

**DOI:** 10.1186/s42494-024-00155-6

**Published:** 2024-05-01

**Authors:** Yinping Li, Xiaoying Hu, Shufang Zhang, Jiani Chen

**Affiliations:** 1 Department of Neurology, West China School of Nursing, Sichuan University/West China Hospital, Sichuan University, Chengdu, 610041 China; 2 Department of Neurology, West China Hospital, Sichuan University, No. 37 Guoxue Road, Chengdu, 610041 China

**Keywords:** Epilepsy, Ictal examination, Video EEG

## Abstract

**Background:**

Ictal examination based on video-based electroencephalography (EEG) is crucial for locating and lateralizing seizures. In this study, we aimed to evaluate the quality of ictal examination in the Comprehensive Epilepsy Center of West China Hospital, Sichuan University, in order to provide information for quality improvement in daily clinical practice.

**Methods:**

Video recordings of 100 patients with epilepsy were retrospectively reviewed. The performance of the ictal examination was independently reviewed by two epileptologists using an ictal examination protocol.

**Results:**

In this retrospective analysis, 589 seizure episodes from 100 patients with epilepsy were reviewed. The ages of the patients ranged from 3 to 77 years, with a mean age of 25.8 ± 12.8 years. Among the 589 seizure episodes, a majority (93.7%) were focal seizures. For 226 (38.4%) seizures, the medical staff arrived at the bedside. Among them, 153 (153/226, 64.7%) seizure episodes, the medical staff arrival at the bedside within 30 s of onset, and 120 (120/226, 53.1%) seizures were tested by the medical staff. The compliance rates for "safety” and "visibility" reached 80% or higher while “naming”, “retelling”, and “memory testing” only reach less than 3%.

**Conclusions:**

Our survey identified the main problems in ictal assessments. It is challenging to complete a standardized examination for new trainees at Epilepsy Monitoring Units. Regularly strengthening training in ictal examination and understanding of semiology may improve patients’ examination ability. However, further study of the implementation of training is necessary.

## Background

Epilepsy manifests as a diverse range of symptoms and signs during seizures, collectively referred to as the seizure semiology [1–3]. Seizure semiology plays a crucial role in determining the location of epileptic seizures [4–6]. In current clinical practice, long-term video electroencephalography (VEEG) is widely used to monitor EEG patterns, clinical symptoms, and signs during epileptic seizures [7, 8]. The recorded videos can be reviewed repeatedly to comprehensively analyze seizure semiology in conjunction with EEG data, providing valuable evidence for clinical intervention and treatment and facilitating the diagnosis and localization of epileptic seizures [9]. However, certain seizure symptoms and signs may be obscured or difficult to detect due to the partial obstruction or limitations in video resolution [10]. Additionally, some seizure-related information may not be directly observable on video and requires physical examination and patient interviews by healthcare providers. For instance, the presence of premonitory symptoms can be determined by evaluating language, cognition, and consciousness and identifying postictal paralysis [11]. Seizure-period examinations and interviews enhance the understanding of seizure semiology, play a significant role in the neuroanatomical localization of epileptic seizures, and provide additional information for epilepsy treatment [12], facilitating early diagnosis and ultimately reducing hospitalization length, medical expenses, and the risk of recurrent seizures [13]. Prolonged generalized epileptic seizures can impair the respiratory function and, in extremely rare cases, lead to death. Therefore, safety measures (such as positioning patients on their side and providing oxygen/suctioning) are an important component of seizure-period assessment [14, 15].

The frequency of long-term continuous VEEG use has been increasing. Jay et al. conducted a survey of 137 doctors from 97 hospitals in the United States, adding data to the current practice of EEG in critically ill adults [16]. However, there is currently no survey or quality control of the practice of ictal examination during seizure episodes in China [13], especially considering the impact of the COVID-19 pandemic [17]. This further hinders the clinical application of long-term video EEG monitoring.

In this study, we retrospectively reviewed the performance of healthcare providers (including doctors, EEG technicians, and nurses) in conducting ictal examinations during the seizure periods of patients by viewing the monitoring videos. The current practice of seizure-period interviews and physical examinations was analyzed, with the aim to improve the standardization and training of seizure-period examinations in the future.

## Methods

### Participants and procedures

This study was carried out at the Comprehensive Epilepsy Center of West China Hospital. We used a standardized, streamlined ictal examination protocol developed previously [12]. The rotary nurses and residents received training before entering the EMU. The inclusion criteria for participants were as follows: (1) patients diagnosed with epilepsy; (2) patients having experienced at least one seizure attack recorded by VEEG. Individuals with no seizures during the VEEG detection or diagnosed as non-epileptic were excluded. From 2021 to 2022, a total of 100 patients who underwent VEEG were consecutively included in this study. The ictal examination performance was rated according to the protocol and the most frequent problems in the performance were identified for further clinical improvement. All patients or their guardians had provided consent to participate in the study. This study was approved by the Ethics Committee of the West China Hospital of Sichuan University (No. 2020–988).

By watching the EEG videos, we counted the duration of seizure episodes, whether the event alarm was pressed during the seizure, the time from the onset of the seizure to the arrival of medical staff at the bedside, whether examination was performed during the seizure, and who performed the examination. The ictal examination consisted of 10 components: (1) safety, i.e., the medical staff arrives at the bedside and ensures the patient's safety and that the airway be unobstructed; (2) visibility, uncovering the patient and ensuring that the patient is in camera; (3) loud description of the clinical manifestations, especially descriptions on subtle and imperceptible manifestations such as manifestations of eyes and the mouth; (4) seizure aura inquiry (e.g., “What do you feel right now?”); (5) memory testing: show the patient a word, such as "blue", and ask he/she to repeat it in the subsequent test; (6) language testing: autonomous language (7) retelling (e.g., "read with me, today is a sunny day"); (8) naming: show patient a pen, and ask he/she what it is; (9) orientation questions (e.g., "Where are you now? What's the date today?”); (10) motor function assessment: check the muscle strength of both limbs. We observed a total of 120 ictal examination videos. The ictal examination was categorized as "Executed" if all examinations were completed with cooperation, and as "Not executed" if the examination was not completed or the patient lacked cooperation.

The clinical fellows and nurses rotated in our EMU for three to six months, ensuring a stable team of health care professionals throughout the study. In addition to providing training before rotation, we distributed an ictal examination cue card to each fellow and nurse, and the ictal examination note was posted in every EMU patient room. We recorded every ictal examination result in a shifting notebook and initiated the practice of discussing patient-specific considerations with staff in daily rounds.

### Statistical analysis

We described the general information of the patients and their ictal examination frequency and percentage, including age, sex, seizure type, whether medical personnel arrived at the bedside, whether they pressed the event alarm, seizure onset time, whether an ictal examination was performed, and who conducted the examination. Furthermore, the time of arrival at the bedside and the compliance with the eleven components of the examination were also analyzed. Data analysis was performed with the SPSS 22.0 (SPSS, Inc., Chicago, IL) software. Categorical data were compared by the chi-squared test or Fisher’s exact probability test as appropriate. Two tailed *P* < 0.05 was considered statistically significant.

## Results

### General information

Videos of 100 ictal patients (55 males and 45 females) with a total of 589 seizure episodes were reviewed from November 2021 to March 2022 by two epileptologists (Jiani Chen and Xiaoying Hu). The age of the patients ranged 3–77 years, with a mean age of 25.8 ± 12.8 years. Of the 589 seizure episodes, a majority (93.7%) were nonconvulsive seizures.

### Execution of seizure ictal examination

Among the 589 seizure episodes recorded, during 312 (53%) seizure episodes, the accompanies of the patients pressed the event alarm button at the occurrence of seizures, and medical staff arrived at the bedside in 226 instances (38.4% of 589). Details of the timing of medical staff arrivals at the bedside are presented in Table 1. Among the medical stuff do arrived episodes, more than half (67.7% of 226) of the episodes, medical staff reached the patient's bedside within 30 s. We compared the time of arrival of the medical staff at the bedside during nighttime and daytime seizures, and medical staffs were statistical more frequently arrived within 30 s in daytime than nighttime (76.5% vs 55.3%, *P* = 0.0008).

**Table 1:** Time of medical staff arrival at the bedside during 226 ictal episodes

Time of arrival at the bedside	Daytime seizures		Nocturnal seizures		total		*P* value
	*n*	Percentage (%)	*n*	Percentage (%)	*n*	Percentage (%)	
≤ 30 s	101	76.5%	52	55.3%	153	67.7%	0.0008
> 30 s	31	23.5%	42	44.7%	73	32.3%	

The examinations during seizure episodes were conducted by 106 individuals, including resident doctors (70.8%), nurses (29.2%). And a majority of them possessed a bachelor's degree or higher. However, approximately 86% had less than 5 years of work experience (Table 2). During 120 episodes, an ictal examination was performed. The compliance rates for "safety" and "visibility" reached 80% or higher. The execution rate of autonomous language testing reached 70%. Approximately 20.8% of the patients were asked for what the aura was before the seizure, and 22.5% of the patients had strength testing However, the medical personnel described the clinical manifestations loudly in only 8.3% of the seizures, and the healthcare professionals performed a memory test in only 1.7% of the seizures. “Retelling” and “naming” accounted for 0.8% and 2.5%, respectively. In the ictal examination, only the execution rate of “autonomous language” and “follow instruction” in the language test showed significant differences between nocturnal and daytime seizure episodes (Tables 3 and 4).

**Table 2:** Basic information of medical staffing in seizure ictal examination (*n* = 106)

		Resident doctors (*n* = 75)	Nurses (*n* = 31)	Total (*n* = 106)
Sex	Male	28	3	31
	Female	47	28	75
Title	Junior level	21	29	50
	Intermediate level	54	2	56
	Senior level	0	0	0
Educational level	College degree	0	3	3
	Bachelor degree	60	28	88
	Master degree	15	0	15
	Doctoral degree			
Work experience	1–2 years	15	17	32
	3–5 years	55	4	59
	6–9 years	5	2	7
	10 years or more	0	8	8

**Table 3:** The ictal examination performance in daytime and nocturnal seizures

Ten aspects of physical examination	Daytime seizures		Nocturnal seizures		total		*P* value
	n	percentage (%)	n	percentage (%)	n	percentage (%)	
Safety	56	77%	41	87%	97	80.8%	0.153
Visibility	55	75%	41	87%	96	80%	0.112
Describe the clinical manifestation loudly	9	12%	1	2%	10	8.3%	0.102
Ask for aura	18	25%	7	15%	25	20.8%	0.199
Memory testing	2	3%	0	0%	2	1.7%	
Language testing							
Autonomous language	60	82%	24	51%	84	70%	0.000
Retelling	1	1%	0	0%	1	0.8%	
Following instruction	56	77%	17	36%	73	60.8%	0.000
Naming	2	3%	1	2%	3	2.5%	1.000
Strength testing	18	25%	9	19%	27	22.5%	0.481

**Table 4:** General information of patients and seizure ictal examination

Variable	Frequencies	Percentage (%)
Patient age (*n* = 100 patients)		
3 – 17	22	22
18 – 77	78	78
Sex (*n* = 100 patients)		
Male	55	55
Female	45	45
Seizure type (*n* = 589 seizure episodes)		
Convulsive seizure	37	6.3
Nonconvulsive seizure	552	93.7
Medical staff arrival at the bedside (*n* = 589 seizure episodes）		
Yes	226	38.4
No	363	61.6
Push the event alarm (*n* = 589 seizure episodes)		
Yes	312	53.0
No	277	47.0
Onset time of seizures (*n* = 589 seizure episodes)		
Day shift (08:00–20:00)	220	37.3
Night shift (20:00–08:00)	369	62.7
Ictal examination (*n* = 589 seizure episodes)		
Yes	120	20.3
No	469	79.7
Staff performance of ictal examinations (*n* = 120 ictal episodes)		
Resident doctor	82	68.3
Nurse	34	28.3
Doctor and nurse	4	3.3

## Discussion

The ictal examinations by medical staff during seizures provide supplemental information on seizure symptoms, which can help improve the accuracy of diagnosis and determine the location of the epileptic focus [10]. In this study, the patients included had a wide range of ages, ranging from 3 to 77 years, which aligns with the characteristic of epilepsy onset occurring at any age [18]. Our study of 589 seizure episodes of 100 patients revealed that a total of 226 episodes (38.4%) were accompanied by medical staff arrival at the bedside during the seizures. Among these, ictal examinations during the seizure were conducted in 120 episodes (53.1%).

At only ~50% of the episodes, the accompanies of patients pressed the event alarm button to notify the medical staff. This may be partly because they missed identification part of seizures, and sometimes because they forgot to press the event alarm button at the emergency due to inadequate instructions. The results of this study also showed that the majority of seizures occurred during the night, which is consistent with the findings of Hoppe et al. [19]. This suggests a need for more effective monitoring and communication systems for ictal patients, especially during the night when patients companions may be less alert [20]. The findings of this study also showed that approximately one-third of the ictal examinations were carried out exclusively by nurses, significantly lower than the number of assessments conducted by resident doctors. However, research has suggested that nurses often play a crucial role as the first responders to seizures, and should be adequately trained in event testing [21]. The lower rate of ictal examinations performed by nurses may be explained that the nurses were occupied with other clinical responsibilities and potentially lacked sufficient proficiency in conducting ictal examinations. As nursing education not only enhances nursing knowledge but also improves nurses' self-efficacy in delivering care to patients who previously felt uncertain about caring for them [22], the implementation of an educational program for nurses in the EMU is necessary.

The low rate of ictal examination at our center was concerning, as only 38.4% of all seizures were attended to by medical personnel at the bedside. This rate was lower than that reported by Kandler et al., which reported a staff participation rate of 56% [23]. The low rate may be a result of the absence of dedicated VEEG staff members monitoring seizures 24 h per day. This inadequacy is prevalent in understaffed epilepsy centers in China. Nurses and resident doctors could only become aware of a seizure occurrence if the patients or their accompanies press the event button or if they happen to witness an episode through the monitoring screen. This study suggests that our center lacks sufficient resources and personnel to effectively address this issue. Furthermore, during the day shift, a single nurse has a workload of caring for 8–10 patients, whereas during the night shift, the work load increases significantly, with one nurse caring for nearly 30 patients. In contrast, centers in Korea, Japan and the US adhere to a more favorable nurse-to-patient staffing ratio, where one nurse is assigned to the care of only 1–4 patients [24–26]. Additionally, during nighttime EEG monitoring, night shift nurses after completing their daytime work have alert fatigue due to their involvement in multiple tasks within the ward, potentially resulting in delayed recognition of calls originating from the nursing station leading to the longer arrive time and lower proformance of ictal examination.

In this study, among the medical staff do arrived episodes (226), medical personnel arrived within 30 s more than half (67.7%) of them, which was consistent with the findings of a previous study [27]. Research has shown that seeking medical attention within 30 s after onset of an epileptic seizure is safe for patients [23]. In addition to facilitating surgical and treatment decision-making by rapidly assessing patients during the seizure period, prompt responses of healthcare providers to patients experiencing epileptic seizures can reduce the risk of seizure-related falls or injuries [28] and decrease the risk of unexplained sudden death [29]. Therefore, based on the current situation of staffing shortage in Chinese epilepsy centers, it is recommended that nurses be provided with portable call devices and specialized training on epilepsy-specific examinations. Additionally, enhancing education and training programs for nurses, specifically focusing on seizure recognition and response protocols, would improve their knowledge and skills in managing seizures. Optimizing nurse-to-patient ratios, establishing a comprehensive communication system, and conducting regular quality assessments could further contribute to the improvement of the rate of ictal examination, the patient safety, and the management of seizures in the healthcare setting.

Another finding of this study is that the compliance rates for some steps of the examination during seizure episodes, such as safety and visibility, were relatively high, while others, such as memory testing and naming, were very low, consistent with a previous study [12]. This may be related to a lack of understanding of the significance and the principles of ictal examination [30, 31]. Healthcare providers often discontinue further examination steps if patients do not respond to questions asking for their names. However, memory testing and naming are more complex and specific steps that can reveal subtle cognitive impairments and lateralize the seizure focus. Therefore, we suggest that medical personnel not only perform the basic steps of the examination during seizure episodes but also try to perform more advanced steps that can provide more valuable information for the diagnosis and management of ictal patients. Furthermore, we propose regular feedback and evaluation of the performance of medical personnel in the examination during seizure episodes to identify their strengths and weaknesses and improve their skills and confidence. In addition, among the healthcare professionals conducting physical examinations during seizure episodes in this study, approximately 86% have less than 5 years of work experience. Consequently, there is a growing need for specialized and targeted training for these individuals in the future. Moreover, fatigue among staff members is also a reason for the low execution rate of examinations during seizure episodes. For patients with a high seizure frequency reaching up to 9 seizures, healthcare providers may have conducted examinations during the initial seizures but not during subsequent seizures.

During epileptic seizures, impaired consciousness can put patients at risk, especially if they are operating dangerous machinery or driving. This risk is particularly high for patients with refractory epilepsy. Studies have shown that shorter intervals between seizures are associated with an increased risk of epilepsy-related motor vehicle accidents [32]. Considering the danger of impaired consciousness for patient safety [33–35], it is essential to assess whether impairment occurs during seizures. Therefore, training on seizure-period examinations, which can be divided into theoretical and practical components, is recommended for personnel at different levels. Theoretical training will inform healthcare providers about the importance of seizure-period examinations and their content and principles. Practical training, on the other hand, will strengthen clinical skills through simulated practice and operational exercises.

Our study has several limitations. First, the sample size in our study was small. Additionally, repeated assessments of the same patient could lead to fatigue of the examiner, potentially introducing bias to the overall results. Second, our study is a retrospective single-center investigation, raising the possibility of recall bias and selection bias. In our study, 93.7% of patients experienced non-convulsive seizures. This may be related to the fact that our retrospective analysis focused on patients who had already been diagnosed with epilepsy and doing VEEG for pre-surgery evaluation. In the future, more prospective studies on the assessment of convulsive and non-convulsive seizures are needed. Furthermore, factors such as the performance changes with time of practice, could influence the evaluation. Unfortunately, our study did not include an analysis of these factors, and future studies should include this analysis to clarify the effects of these variables.

## Conclusions

This study was the first to report the execution of ictal examination of seizures in China. A majority of the medical personnel arrived at the patient's bedside within 30 s, and the execution rate of safety and visibility aspects of physical examination was high, but language and memory aspects were low. We found that there is a need for further improvement in the promptness of medical staff arrival at the bedside and the standardization of physical examinations during seizure periods. It is highly essential to incorporate seizure semiology, seizure period history and ictal examination data into routine training to improve the execution rate of seizure-period examination. Further multicenter studies with larger sample sizes are needed. Additionally, further investigations are warranted to explore the correlations between seizure types and the execution of seizure-period examinations, as well as the effectiveness of systematic training.

## Data Availability

All the data generated or analyzed during this study are included in this published article.

## Abbreviations

EEG: Electroencephalography
EMU: Epilepsy monitoring unit
VEEG: Video electroencephalography
